# A Case of Retroflexion of the Uterus Treated by Daily Replacement and the Supporting Tampon

**Published:** 1886-02

**Authors:** Virgil O. Hardon

**Affiliations:** Atlanta, Ga.


### A CASE OF RETROFLEXION OF THE UTERUS TREATED BY DAILY REPLACEMENT AND THE "SUPPORTING TAMPON."

BY VIRGIL O. HARDON, M. D., ATLANTA, GA.

The following case is reported, not because it is in any degree rare or remarkable, but because it illustrates a method of treatment which has, in my hands, yielded more satisfactory results than any other. Retroflexion of the uterus is one of the most frequent of all pelvic derangements, and at the same time one of the most unsatisfactory, both to the physician and patient, as regards the results of treatment. Any effort, therefore, to throw new light upon the subject needs no apology.

E. G., colored, aged about thirty, unmarried, has never been pregnant. Menstruation began at the age of thirteen, and until the present attack has always been regular, but accompanied by a considerable degree of pain. General health has always been good heretofore. Three years ago she sustained a severe fall, striking upon her buttocks. From that time on the following symptoms were developed and gradually increased in intensity, viz.: Pain in the back and loins, difficulty of locomotion, dysmenorrhœa, painful defecation, incontinence of urine, loss of strength and appetite, emaciation. Since June, a period of four months, her menses have been entirely absent. For six months she has been unable to work, and has been dependent upon the charity of her friends. Her urine dribbles from her constantly during the day, and at night she is compelled to rise every half hour to void it. The annoyance and loss of sleep arising from this condition, together with her constant, severe pains, render her life a burden.

She was first seen by me October 29, 1885, at which time vaginal examination showed a marked retroflexion of the uterus at the internal os, the fundus projecting backward and encroaching upon the rectum, and the cervix pointing forward in the direction of the axis of the vagina. The whole organ was enlarged and tender and was low down in the pelvis, the os being within an inch of the vulva, but was freely movable. The roof of the pelvis was tender with some hardness in the tract of the utero-sacral ligaments. The anterior wall of the vagina was partially prolapsed. The cervix was long and conical and the external os so small that it was necessary to slit it bilaterally with a bistoury before Simpson's sound could be introduced. The sound passed directly backward after entering the womb and showed a depth of two and a half inches. Rectal examination confirmed the diagnosis of retroflexion and showed the body of the womb resting against the rectum low down in the pelvis. The ovaries could not be detected. A catheter inserted into the bladder showed the dribbling of urine to be due to incontinence and not to over-distention.

After slitting the external os bilaterally, a Simpson's sound was introduced to the fundus, and by slow and steady rotation, aided by a finger in the rectum, the womb was brought to its normal position of slight anteflexion. With the right index finger in the vagina and without removing the sound, the organ was then raised to its normal elevation in the pelvis. The "supporting tampon" was then introduced, consisting of a pledget of cotton saturated with glycerine, in the posterior cul-de-sac, another in the anterior fornix, and a third and fourth on either side of the cervix, with a larger pledget placed between the cervix and the vaginal promontory. The patient was then directed to stand, and it was found that by the tampon the womb was held at its normal elevation in the pelvis, and the vaginal prolapse no longer existed. The patient declared herself to be more free from pain than she had been for months.

The replacement of the womb and the insertion of the "supporting tampon" were repeated daily for thirty-two days. No other treatment was used. At the end of a week all pain had disappeared. The patient was able to hold her urine for an hour at a time during the day, and was obliged to rise only once in the night to void it. Pain upon defecation had ceased. She had gained in appetite and in flesh, and, to use her own expression, she "felt like a new woman." She was able without discomfort to walk to my office, a distance of over a mile. The improvement was rapid and steady, and at the end of thirty-two days the treatment was stopped. The womb was found to be of normal size and void of tenderness, and remained in its proper position without support. The patient had complete control of the bladder, and was free from all pain. In a word, she was well, and she resumed her occupation of cook for a family of sixteen persons. Her menses, however, had not returned. On the first of December, therefore, having stopped all other treatment, I prescribed permanganate of potash internally, in doses of five grains three times a day, as recommended by Ringer and Murrell. After taking this remedy for nineteen days, the menses appeared in normal quantity and without pain, lasting four days. The patient has been seen at intervals of one week since that time, and, although she is subjected to hard work for twelve hours every day, there has been no return of her former symptoms.

